# The Role of Hyperglycemia and Insulin Resistance in the Development and Progression of Pulmonary Arterial Hypertension

**DOI:** 10.1155/2016/2481659

**Published:** 2016-06-08

**Authors:** Daniel Grinnan, Grant Farr, Adam Fox, Lori Sweeney

**Affiliations:** ^1^Division of Pulmonary and Critical Care Medicine, Virginia Commonwealth University Health System, Richmond, VA 23298, USA; ^2^Department of Internal Medicine, Virginia Commonwealth University Health System, Richmond, VA 23298, USA; ^3^Division of Endocrinology, Virginia Commonwealth University Health System, Richmond, VA 23298, USA

## Abstract

Pulmonary hypertension is a progressive disorder which often leads to right ventricular failure and death. While the existing classification system for pulmonary hypertension does not account for the impact of diabetes mellitus, evidence is emerging that suggests that diabetes is associated with pulmonary hypertension and that diabetes modifies the course of pulmonary hypertension. There is also growing radiographic, hemodynamic, biochemical, and pathologic data supporting an association between diabetes and pulmonary hypertension. More robust epidemiologic studies are needed to confirm an association between diabetes and pulmonary hypertension and to show that diabetes is a disease modifier in pulmonary hypertension. In addition, evaluating the effects of glucose control in animals with pulmonary hypertension and diabetes (as well as in humans) is warranted.

## 1. Introduction

Pulmonary hypertension (PH) refers to an abnormally elevated blood pressure in the pulmonary circulation that can lead to right ventricular (RV) failure and death [1]. Interestingly, PH has been partitioned and separated from other vascular disorders, including systemic hypertension. Therefore, while the role of diabetes mellitus in the pathogenesis of systemic microvascular and macrovascular disease has been appreciated for decades, there has been little evaluation of the potential role that diabetes could have in the pathogenesis of PH. The existing classification of PH does not account for the potential influence of diabetes or other components of the metabolic syndrome, and current treatment is focused on the use of pulmonary vasodilators. Only recently have we begun to understand that not only diabetes may predispose to PH, but also it may fundamentally alter the prognosis in those with PH. Below, we will review the current diagnosis and management of PH, the clinical evidence supporting a role of diabetes in the pathophysiology of PH, the biochemical evidence suggesting a role of local hyperglycemia and insulin resistance in the development of PH, and directions for future research.

## 2. Current Classification and Treatment of Pulmonary Hypertension

Since 1996, there has been a classification system developed by the World Health Organization (WHO) and comprising 5 groups [2]. This system has been subject to minor changes over time, but it has remained relatively stable (Table 1).

Group I is pulmonary arterial hypertension (PAH) and is characterized by progressive obliteration of the pulmonary arterioles due to endothelial and smooth muscle proliferation [3]. Several underlying conditions (connective tissue disease, HIV infection, congenital heart disease, portal hypertension, genetic polymorphisms, and anorexigen or amphetamine use) are known to predispose to PAH. PAH patients have a varying course and prognosis depending on how their right ventricle tolerates the increased afterload [4]. Group II is pulmonary venous hypertension (PVH) occurring when a left heart condition (systolic, diastolic, or valvular dysfunction) leads to an increase in pulmonary arterial pressure. This is the most common type of PH [5]. Over time, the pulmonary vascular bed can be chronically and permanently changed. Group III is pulmonary hypertension related to chronic hypoxia. This can be from untreated obstructive sleep apnea or from a variety of severe lung diseases (emphysema, pulmonary fibrosis, and bronchiectasis). Group IV is chronic thromboembolic pulmonary hypertension (CTEPH), a condition resulting from unresolved pulmonary thromboembolic disease. CTEPH may require medical or surgical management [6]. Group V is miscellaneous, implying that the underlying physiology and treatment is poorly understood. Group V includes those patients with PH associated with end stage renal disease, sarcoidosis, sickle cell disease, and others. Patients are classified into these “groups” to optimize the chance that pulmonary vasodilators will be effectively used. Patients with PAH are generally treated with pulmonary vasodilators, while patients with groups II or III disease are not known to respond to pulmonary vasodilators. However, there are many group II/III patients who have “pulmonary hypertension out of proportion” to their underlying condition, and it is not established how to treat these patients [1].

Pulmonary vasodilator therapy is expensive and complicated [7]. At the present, there are 13 pulmonary vasodilator therapies approved for use in the United States. However, each therapy falls into one of 3 biochemical pathways. The first approach activates cyclic GMP through the nitric oxide pathway. This group included the phosphodiesterase type 5 inhibitors and the soluble guanylate cyclase agonists. The second approach includes endothelin receptor antagonists, a group of medications that block a potent vasoconstrictor (endothelin) from activating its receptor. The third approach targets the prostacyclin pathway, as prostacyclin causes intense vasodilatation in the pulmonary vascular bed when bound to its receptor. In clinical practice, it is common for patients to be on combination therapy [1, 8], where vasodilators targeting different pathways are combined. Pulmonary vasodilator therapy, when combined with management of right ventricular failure and optimization of oxygen delivery, has resulted in improved patient outcomes. However, PH remains a deadly disease [9].

## 3. Clinical Impact of Hyperglycemia and Glucose Intolerance on the Development and Progression of Pulmonary Hypertension

The above framework for the classification of PH and management of PAH does not mention a potential role for hyperglycemia or glucose intolerance, as there has been insufficient evidence to link the conditions (hyperglycemia and glucose intolerance) with the disease (PH). However, there is evidence that diabetes is an independent predictor (OR  1.53, *p* < 0.001) for the development of PH, even after other components of the metabolic syndrome are controlled for. In addition, an abnormally high percentage of patients are found to have glucose intolerance at the time they are diagnosed with PAH. Four pulmonary hypertension centers have systematically assessed those patients newly diagnosed with pulmonary hypertension for diabetes mellitus [10–12]. When the experiences from these centers are combined, a total of 415 PH patients were evaluated, of whom 107 (26%) had diabetes. This correlates with data from the UK and Ireland pulmonary hypertension registry, which found that 23% of PH patients over the age of 50 had diabetes [13]. While these studies provide only a snapshot of the PH community, the incidence of diabetes in the PH population appears to be higher than the incidence of diabetes in the general population over the age of 45 (19%) [14] and suggests a connection between diabetes and PH.

In those already diagnosed with PH, diabetes appears to have a significant impact on their disease course. It has been well established that current patients with pulmonary hypertension are older (average age 53.1 in the REVEAL cohort) [15] and have more comorbidities compared with cohorts from the 1980s and 1990s (average age 36 in the NIH cohort) [16]. Not surprisingly, the older PH population is much more likely (*p* < 0.001) to have diabetes compared with the younger PH population [13]. There are now several single-center or two-center studies that have found worse survival in patients with PH and diabetes, compared with those PH patients without diabetes. One showed that, at the time a patient is diagnosed with PH, hemoglobin A1C less than 5.7 was an independent predictor of survival (*p* < 0.002) [17]. A separate analysis found that patients with PH and diabetes had worse survival (hazard ratio 1.7, *p* = 0.04) compared with other PH patients [18]. A third study found that 10-year survival was worse in those with diabetes and PH compared with those without diabetes (*p* = 0.04) [12]. While these studies only show an association and cannot show that diabetes leads to PH and accelerates the disease, they do raise the question “why would the presence of diabetes be harmful to those with PH?” To answer this question, we will look at existing research that suggests potential involvement of the microvascular circulation of the pulmonary arterioles and the right ventricle as related to diabetes.

## 4. Right Ventricular Failure and Diabetes Mellitus

Many clinical studies have shown that the prognosis of patients with PH is dependent on the right ventricle's ability to tolerate the increased afterload imposed by pulmonary hypertension. In patients with PH, the RV is resultantly hypertrophied and enlarged. The hypertrophied RV is subject to ischemia, and this ischemia is associated with RV dysfunction and prognosis [19]. Ischemia may be related to increased afterload, to increased myocardial density without a compensatory increase in right ventricular angiogenesis, and to RV microvascular injury impairing oxygen delivery. In addition, it has been well documented that patients with systemic sclerosis have increased right ventricular fibrosis, and the resultant impairment in their RV function is linked to a poor prognosis. Thus, both RV ischemia and RV fibrosis impact the prognosis of patients with PH.

Several imaging studies have suggested that diabetes affects the RV. Cardiac magnetic resonance (CMR) and 3-dimensional echocardiography have emerged as tools that provide important information about the right ventricle. Imaging studies utilizing these techniques have found that right ventricular end-diastolic volume is reduced in patients with diabetes (after controlling for other potential risk factors) [20, 21] and that right ventricular stroke volume is impaired in patients with diabetes and PH (without a change in pulmonary vascular resistance) [12]. These imaging studies correlate with existing hemodynamic data. Patients with PH and diabetes (PH-DM), when compared to patients with PH alone, have repeatedly been found to have higher right atrial pressure despite having a trend toward lower pulmonary arterial pressures [10] and pulmonary vascular resistance [11]. In the AMBITION study, the PH-DM cohort has a worse 6-minute walk distance (a surrogate for RV function in patients with PAH) compared to a matched PH cohort, despite a trend toward lower pulmonary arterial pressures [22]. These results have led to acceptance that patients with PH-DM and other features of the metabolic syndrome behave differently than patients with PH alone, and recent clinical studies have amended their exclusion criteria so that these patients are excluded from participation [23]. While further research is needed, the above radiographic and hemodynamic data suggests that the right ventricle is adversely impacted by diabetes in patients with PH and that RV dysfunction leads to fundamental differences between cohorts of patients with pulmonary arterial hypertension.

**Figure 1:**
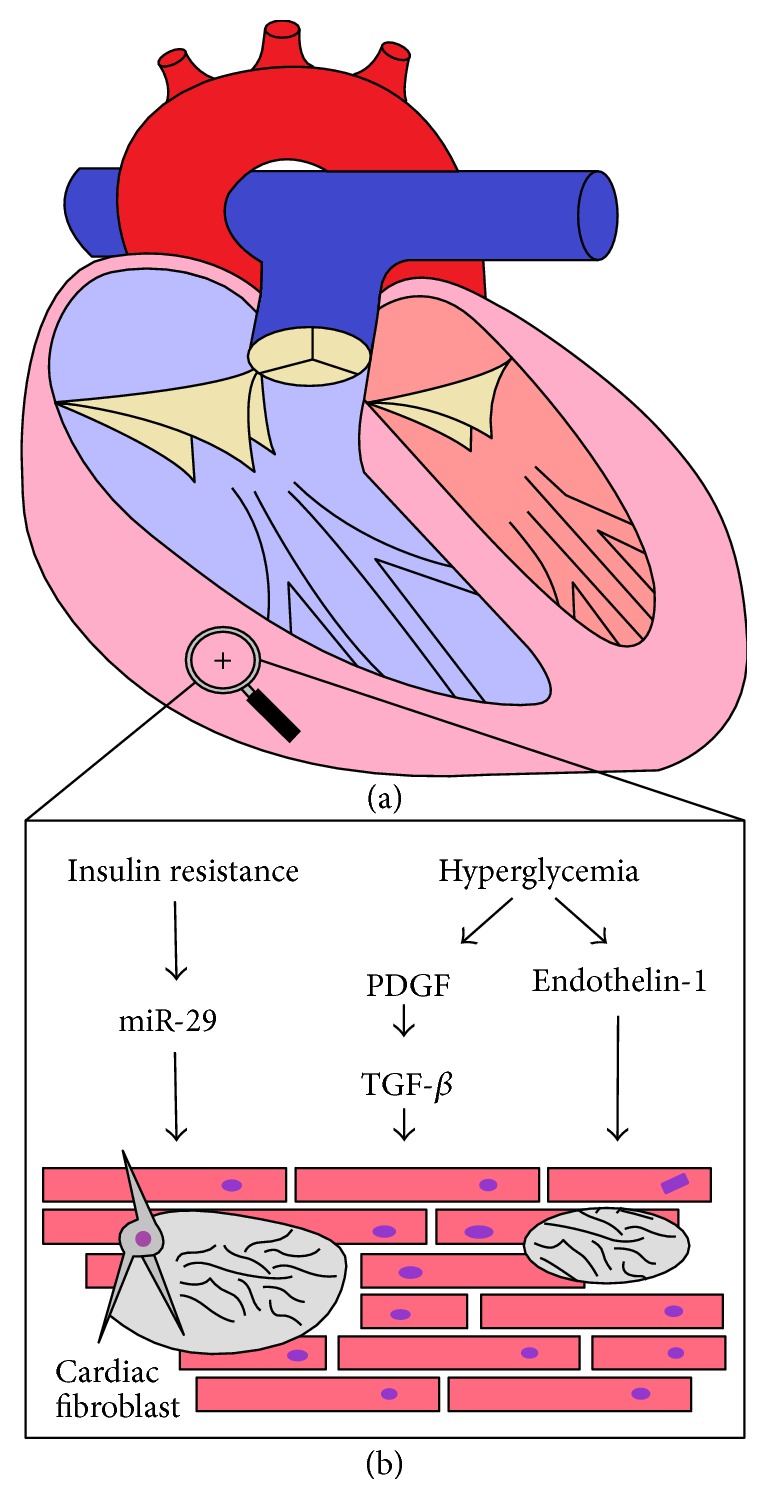
(a) Depiction of a heart with right ventricular hypertrophy and fibrosis. (b) Summary of the known pathways connecting diabetes to RV fibrosis and hypertrophy.

The above recognition that patients with PH-DM have physiologic differences in their RV function (compared to those with PH alone) suggests that diabetes may impair the right ventricle in patients with PH-DM by predisposing to fibrosis, ischemia, or both. There are several established biochemical pathways that predispose the RV to fibrotic changes in patients with diabetes (Figure 1).

Platelet derived growth factor (PDGF) is upregulated by local hyperglycemia, in turn increasing transforming growth factor-B (TGF-B), which is profibrotic [24]. TGF-B plays a major role in the fibrosis caused by diabetes in other organs, such as the kidney and the left ventricle [25, 26]. Regional hyperglycemia also causes another profibrotic pathway, induced by endothelin-1, to be activated [27]. Increased levels of endothelin-1 have been linked to patients with PH for many years through its vasoconstrictive effect on the pulmonary arterioles, but the profibrotic role of endothelin-1 in the RV remains poorly understood. In addition, insulin resistance leads to upregulation of the diabetic marker microRNA miR-29 family, which causes the cardiac fibroblast to increase collagen production and myocardial fibrosis [28]. Therefore, it is no surprise that, when myocardial biopsies from the right ventricle of patients with diabetes have been compared with controls without diabetes, there is an increase in RV fibrosis in those with diabetes [29]. While the role of fibrosis in causing left ventricular cardiomyopathy in patients with diabetes has been well established for many years [30], the role of fibrosis in right ventricular failure of patients with PH-DM is just emerging. The above findings suggest that, as more is learned about the role of hyperglycemia and insulin resistance specific to the RV in patients with diabetes, we will find that fibrosis plays a major role in RV dysfunction and clinical outcomes.

RV ischemia is also likely to contribute to the decreased RV function in patients with PH-DM. Capillary rarefaction, defined as a decrease in the density of myocardial arterioles within the right ventricular myocardium, is established in PAH [31]. As the maladaptive RV myocardium has decreased not only capillary density to deliver oxygen to the myocardium but also myocardial hypertrophy and fibrosis, RV ischemia is a common problem in patients with PAH [19]. The symptom of exertional, substernal chest pressure in patients with PAH has also been linked to elevated troponin T levels, a marker of RV ischemia [32]. In patients with PAH who develop RV ischemia (as evidenced by elevated troponin T), survival is worse, thus highlighting the importance of ischemia in this disease [33]. While no existing studies have evaluated the role of diabetes in creating RV ischemia in patients with PH-DM, the role of diabetes in promoting ischemia in other vascular beds (including the left ventricle) [34] is well established.

## 5. Diabetes Mellitus and the Pulmonary Microvasculature

Pulmonary arterial hypertension is a disease of the pulmonary arterioles, where there is proliferation of endothelial cells and smooth muscle cells, eventually leading to vascular narrowing or even obliteration. There are many established mediators that contribute to the vasoconstriction and pulmonary arteriolar proliferation that characterize PAH. As mentioned above, nitric oxide and prostacyclin are potent vasodilators of the pulmonary circulation. They also inhibit endothelial proliferation within the pulmonary arterioles. Endothelin is a potent vasoconstrictor and mitogen in the pulmonary circulation and it is overexpressed in patients with PAH [35]. In addition to these chemical mediators, mutations in the bone morphogenetic protein receptor 2 (BMPR2) gene have been associated with both familial and idiopathic PAH [36, 37]. These mutations lead to decreased BMPR2 activity within the smooth muscle cells of patients with PAH, and this in turn leads to the overexpression of transforming growth factor-B (TGF-B) and increased smooth muscle and myofibroblast proliferation. Insulin like growth factor (ILGF-1), a mitogen for pulmonary arteriolar smooth muscle proliferation, is locally upregulated in the smooth muscle cells of PAH patients [38]. Last, peroxisome proliferator-activated receptor gamma (PPAR*γ*), a transcription factor which is antiproliferative and proapoptotic, is decreased in pulmonary hypertension and contributes to endothelial proliferation [39].

**Figure 2:**
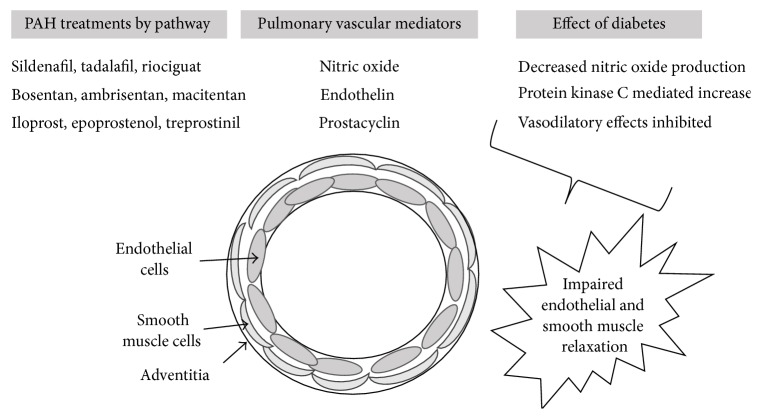
Role of diabetes mellitus in promoting vasoconstriction within the pulmonary arteriole and the potential impact on the efficacy of medications commonly used to treat pulmonary hypertension.

Local hyperglycemia and insulin resistance influence all of the above pathways that have been implicated in the development of PAH. Hyperglycemia inhibits endothelial nitric oxide synthase (eNOS), thereby decreasing production of nitric oxide within the endothelial cell [40]. In addition, hyperglycemia generates reactive oxygen species [41], which decrease nitric oxide bioavailability independent of eNOS regulation [42]. Hyperglycemia also activates protein kinase C (PKC) within the endothelial cells, which further decreases nitric oxide production while increasing endothelin levels, TGF-B levels, and inflammatory mediators (NADPH and NF-kB) [43]. In addition, activation of PKC inhibits the vasodilatory effect of prostacyclin (Figure 2) [44].

Insulin resistance was developed in Apo E deficient mice fed a high fat diet, and these mice developed PH that was ameliorated with rosiglitazone induced activation of PPAR*γ* [45]. This study suggests that insulin resistance reduces PPAR-*γ* levels, which predisposed the animals to PH. Another intriguing study found that mice deficient in BMPR2 developed insulin resistance [46]. Moreover, if these mice were fed a high fat diet, the penetrance of PAH was increased. All of the above data suggests that insulin resistance and/or hyperglycemia may influence the known molecular pathways involved in the development of PAH, thus providing a physiologic rationale for the increased incidence of diabetes in the PH population that was discussed earlier.

It is also important to note that diabetes has a significant effect on other vascular beds in the body at the level of the capillary bed. Similarly, there is evidence that regional hyperglycemia and resultant oxidative stress increases pulmonary capillary permeability [47]. Therefore, it is possible that damage to the pulmonary vascular system at the level of the capillary bed, in addition to the above-mentioned pulmonary arteriolar involvement, may influence how patients with PH-DM respond to targeted therapy of the pulmonary arterioles.

## 6. Conclusion

The pulmonary vascular bed is one of the only vascular beds in the body where the effects of diabetes have not been well studied. The current classification system (see Table 1) for pulmonary hypertension does not incorporate diabetes mellitus. However, evidence is emerging that suggests not only that diabetes is associated with PH, but that diabetes also modifies the course of PH in patients who have PH-DM. This is suggested by small epidemiologic studies, by hemodynamic studies, by imaging and pathology studies of the RV, and by a growing number of molecular and biochemical studies showing that the determinants of PAH (endothelial cell and smooth muscle cell proliferation) and RV failure (ischemia and fibrosis) are influenced by hyperglycemia and insulin resistance. The existing evidence supports the role for further research in this field. More robust epidemiologic studies are needed to confirm an association between diabetes and PH and to show that diabetes is a disease modifier in PAH. In addition, evaluating the effects of glucose control in animals with PH-DM and in humans with PH-DM is warranted. After all, why would the pulmonary vasculature be selectively spared from the effects of diabetes?

## Figures and Tables

**Table 1:** Current clinical classification of pulmonary hypertension.

Group	Associated conditions
(1) Pulmonary arterial hypertension	Idiopathic, heritable, and connective tissue diseases, congenital heart diseases, drug and toxins, portal hypertension, and schistosomiasis

(1′) Diseases affecting pulmonary capillaries or pulmonary venules	Pulmonary venoocclusive disease or pulmonary capillary hemangiomatosis

(2) Pulmonary venous hypertension	Left ventricular systolic or diastolic dysfunction or left sided valvular heart disease

(3) PH due to lung disease or hypoxemia	Sleep disordered breathing, chronic altitude exposure, chronic obstructive lung disease, and interstitial lung disease

(4) Chronic thromboembolic pulmonary hypertension	

(5) PH due to multifactorial mechanisms	Sarcoidosis, hematologic disorders, chronic renal failure, and glycogen storage disease
